# William F. Wegge, M. D., D. D. S.

**Published:** 1891-06

**Authors:** 


### Editorial, Etc.

WILLIAM F. WEGGE, M. D., D. D. S.—Dr. William F. Wegge, who has just been appointed superintendent of the Northern Hospital for the insane, is a young man, 30 years of age. He is a native of Wisconsin, and was for little more than a year assistant superintendent under Dr. Booth. He graduated from the University of Maryland, Dental Department in 1885, and from the University of Maryland, school of medicine, in 1886, and immediately went to Europe where he attended lectures at the universities of Berlin and Wurzburg. He has made a specialty of insanity and is in every way qualified for the position to which he has been appointed. At the conclusion of his attending lectures in Europe Dr. Wegge returned to the United States and practiced medicine very successfully at Waterford. While in Europe he met Dr. Senn, of Milwaukee. He was a fellow student of Judge Belden, of Racine, at Rochester seminary.

